# Hypoxia-Inducible Factor (HIF) in Ischemic Stroke and Neurodegenerative Disease

**DOI:** 10.3389/fcell.2021.703084

**Published:** 2021-07-28

**Authors:** Elena V. Mitroshina, Maria O. Savyuk, Evgeni Ponimaskin, Maria V. Vedunova

**Affiliations:** ^1^Department of Neurotechnologe, Institute of Biology and Biomedicine, National Research Lobachevsky State University of Nizhni Novgorod, Nizhny Novgorod, Russia; ^2^Department of Cellular Neurophysiology, Hannover Medical School, Hanover, Germany

**Keywords:** hypoxia-inducible factor, HIF, ischemia, hypoxia, adaptation, Alzheimer’s disease, Parkinson’s disease, neurodegeneration

## Abstract

Hypoxia is one of the most common pathological conditions, which can be induced by multiple events, including ischemic injury, trauma, inflammation, tumors, etc. The body’s adaptation to hypoxia is a highly important phenomenon in both health and disease. Most cellular responses to hypoxia are associated with a family of transcription factors called hypoxia-inducible factors (HIFs), which induce the expression of a wide range of genes that help cells adapt to a hypoxic environment. Basic mechanisms of adaptation to hypoxia, and particularly HIF functions, have being extensively studied over recent decades, leading to the 2019 Nobel Prize in Physiology or Medicine. Based on their pivotal physiological importance, HIFs are attracting increasing attention as a new potential target for treating a large number of hypoxia-associated diseases. Most of the experimental work related to HIFs has focused on roles in the liver and kidney. However, increasing evidence clearly demonstrates that HIF-based responses represent an universal adaptation mechanism in all tissue types, including the central nervous system (CNS). In the CNS, HIFs are critically involved in the regulation of neurogenesis, nerve cell differentiation, and neuronal apoptosis. In this mini-review, we provide an overview of the complex role of HIF-1 in the adaptation of neurons and glia cells to hypoxia, with a focus on its potential involvement into various neuronal pathologies and on its possible role as a novel therapeutic target.

## Introduction

Tissue oxygen content plays crucial roles in maintaining the normal functioning of cells and regulation of their development. Reduction in oxygen availability (hypoxia) has different effects on the body depending on the tissue, exposure intensity and duration. Prolonged and pronounced hypoxia results in cellular dysfunction and death. On the other hand, hypoxia can also activate molecular pathways in multiple stem cell systems, including neural stem cells, maintaining their state of differentiation and protecting their DNA from oxidative damage (Mohyeldin et al., 2010).

The central nervous system (CNS) is most sensitive to a lack of oxygen. Although the human brain constitutes only a small portion of the body weight (about 2%), it is the leading consumer of energy. The CNS accounts for over 20% of the total oxygen metabolism, with neurons consuming 75–80% of the total CNS energy usage (Hyder et al., 2013). This energy is primarily required to maintain synaptic transmission and to restore neuronal membrane potentials after depolarization (Harris et al., 2012). Other neuronal functions, including synaptic vesicle recycling, neurotransmitter synthesis, and axonal transport also require energy. Increased neuronal energy consumption is accompanied by an increased intensity of oxygen metabolism in neurons (Rangaraju et al., 2014; Pathak et al., 2015).

The physiological partial pressure of oxygen varies among different regions of the brain. In rats, it fluctuates within the range of 2.53–5.33 kPa in the cerebral cortex, in the range of 1.47–2.13 kPa in the hypothalamus, and is equal to 2.67 kPa in the hippocampus. Under pathological conditions, areas may exhibit severe hypoxia (partial oxygen pressure of < 0.1 kPa) or anoxia (Liu et al., 1995; Erecińska and Silver, 2001). Neurons in the CNS are particularly sensitive to hypoxia, and hypoxia-induced changes of oxygen metabolism and mitochondrial functions can result in destructive structural and functional changes in neurons, leading to their death (Azevedo et al., 2020; Kumari et al., 2020). Ischemic stroke is a widespread disease, and is the main source of hypoxic brain stress (Ratan et al., 2007; Virani et al., 2020). Hypoxia can also be a pathological component associated with neurodegenerative disorders, such as Alzheimer’s and Parkinson’s diseases (Yagishita and Hirasawa, 2017; Snyder et al., 2017).

Even transient ischemic hypoxia can cause severe brain damage, especially when it occurs in the hippocampus, which is extremely sensitive to hypoxia (Gordan et al., 2012). Hypoxia-mediated apoptosis, necrosis, and necroptosis of hippocampal tissue is a main cause of neurological deficits. Since the hippocampus is critical for spatial learning and memory (Simões Pires et al., 2014), damage in this area results in a pronounced decrease in cognitive functions, which seriously reduces the patient’s quality of life (Ünal-Çevik et al., 2004; Lagali et al., 2010; Yang R. et al., 2017; Yang X.S. et al., 2017). Within a few minutes after exposure to cerebral ischemia, a necrotic nucleus of irreversibly damaged cells is formed. This is surrounded by an area of less-affected cells, known as the ischemic penumbra. Cell death in the penumbra is mainly apoptotic, and develops several hours or days after the onset of ischemic stroke (Wang et al., 2017). Therefore, the protection and/or rescue of highly vulnerable cells in the penumbra area is a main therapeutic aim during stroke treatment (Ratan et al., 2007; Broughton et al., 2009). The main methods of preserving the viability of damaged areas involve suppressing processes that lead to neuronal death, and inducing neuronal regeneration.

One of the main regulators of the cell’s response to hypoxia is a protein called hypoxia-inducible factor-1 (HIF-1), which controls the expression of over 700 various target genes that mediate both adaptive as well as pathological processes (Semenza, 2004; Dengler et al., 2014; Barteczek et al., 2017; Wu et al., 2019). The main targets of HIF-1 include genes associated with angiogenesis and energy metabolism. In particular, HIF-1 modulates the expressions of the genes encoding erythropoietin (EPO) and vascular endothelial growth factor (VEGF), and genes that are involved in glucose transport or glycolysis, e.g., glucose transporter-1 (GLUT1), pyruvate dehydrogenase kinase 1 (PDK1), and lactate dehydrogenase A (LDHA) (Leu et al., 2019).

**FIGURE 1:**
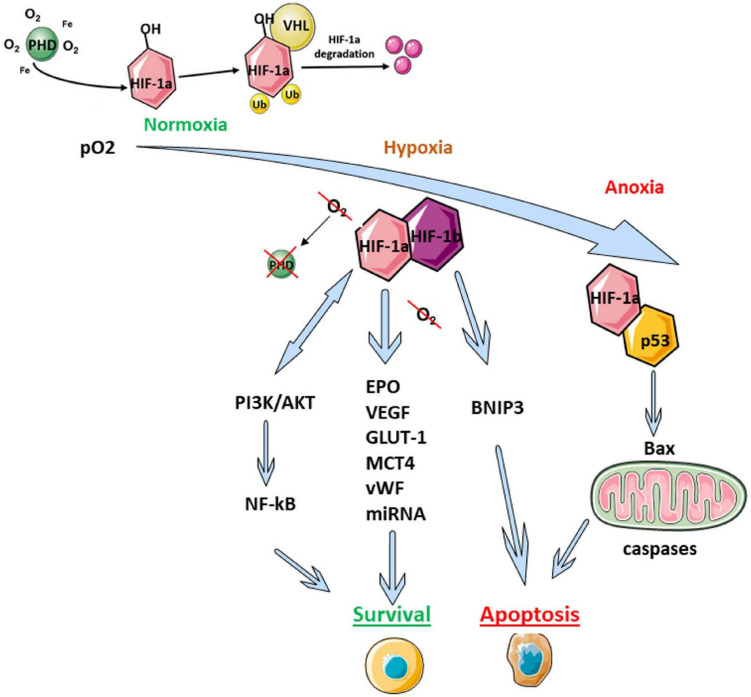
Scheme of HIF-mediated regulation of adaptive cell reactions. AKT1, the serine-threonine protein kinase AKT1; Bax, apoptosis regulator protein, also known as bcl-2-like protein 4; BNIP3, a member of the apoptotic Bcl-2 protein family; EPO, erythropoietin; GLUT-1, facultative glucose transporter 1; HIF, hypoxia-inducible factor; MCT4, monocarboxylate transporter 4; p53, pro-apoptotic transcription factor; PI3K, phosphoinositide 3-kinase; PHD, HIF prolyl hydroxylase; Ub, ubiquitin; VEGF, vascular endothelial growth factor; VHL, von Hippel–Lindau protein; vWF, von Willebrand factor.

HIF-1 is a heterodimer comprising α and β subunits. Under normoxic conditions, the β subunit is constitutively expressed in the cell, while the α-subunit undergoes rapid ubiquitin-dependent proteasome degradation due to the action of oxygen-dependent HIF prolyl hydroxylase (PHD) (Figure 1; Semenza, 2004, 2009). Under hypoxic conditions, PHD is inactivated, leading to stabilization of HIF-1α, followed by its translocation into the nucleus, where it forms a heterodimeric complex with HIF-1β. This complex interacts with DNA and activates the expressions of multiple target genes encoding proteins that help increase the tissue’s oxygen supply by boosting erythropoiesis and angiogenesis (Figure 1). In the CNS, HIF complex activation initiates a neuroprotective response, resulting in restoration of cellular functions that are affected by hypoxia (Bergeron et al., 2000; Ivan et al., 2001; Cho et al., 2014; Semenza, 2014). Inactivation of HIF-1α subunit expression (e.g., by a mutation in the corresponding gene) leads to a more obviously pronounced impairment of learning and decreased neurogenesis during the postischemic period (Carrica et al., 2019). Additionally, inactivation of HIF-1α leads to increased brain damage and decreased survival following ischemia (Baranova et al., 2007; Milosevic et al., 2007; Sheldon et al., 2009).

The HIF-1 signaling pathway generally constitutes a major molecular cascade transmitting signals from multiple factors associated with hypoxic-ischemic brain injury. In this respect, HIF-1 appears to be an universal switch modulating the activities of molecules that control cell survival, glucose metabolism, and metabolic adaptation. HIF-1α has complex effects on the brain, which largely depends on the time-point after hypoxic damage. At the earliest post-ischemic stage (i.e., within 24 h), HIF-1α accumulation promotes cell death (Cheng et al., 2014; Barteczek et al., 2017). In contrast, during the later stage (i.e., > 4 days), HIF-1α signaling has a pro-survival effect through limitation of the infarct size (Baranova et al., 2007; Li et al., 2019).

Multiple studies have also demonstrated HIF involvement in other signaling pathways regulating hypoxic response. For example, HIF induces the expression of hypoxia-sensitive microRNAs (miRNAs) that play an essential role in modulating the cellular response to hypoxic stress (Chen et al., 2013; Zhang et al., 2020). MicroRNAs are a class of small non-coding RNAs that regulate mRNA stability and translation by binding to the 3′UTR, thus leading to degradation of the targeted mRNA, resulting in decreased levels of the corresponding proteins. HIF-1α can reportedly regulate miRNAs at the transcriptional level (Yang et al., 2018). On the other hand, HIF expression can also be regulated by miRNAs, and about 40 miRNAs have been identified as associated with HIF-1α (Serocki et al., 2018). Since the most studies of HIF-linked miRNAs have focused on cancer cell lines, the roles of miRNAs in other cell types is not completely understood (Serocki et al., 2018).

HIF-1α is also involved in regulating the PI3K/Akt pathway, which can modulate the activity of transcription factor NF-κB (Figure 1; Walmsley et al., 2005; Lee J.C. et al., 2016; Kim et al., 2017; Liu et al., 2019). This pathway plays a pivotal role in maintaining cell viability and apoptosis regulation.

Moreover, HIF-1 is known to modulate mitochondrial biogenesis and oxygen consumption across multiple phyla. For example, in renal carcinoma cells HIF-1 negatively regulates mitochondrial biogenesis via inhibition of transcriptional activity of transcriptional factor c-Myc by the expression of MAX-interacting protein 1 (MXI-1) inhibiting the Myc transcriptional activity and increased c-Myc degradation by the proteasome (Zhang H. et al., 2007). As consequence, c-Myc-mediated expression of the mitochondrial transcription factor A (TFAM), which encodes a key factor involved in mitochondrial transcription and mitochondrial DNA replication, is decreased leading to reducing the mitochondrial activity. Similar mechanism was obtained in insect (Lin et al., 2016). The work by Lin et al. (2016) demonstrated that during diapause, the level of HIF-1a in insect pupae is increased, leading to a significant decrease in the levels of c-Myc and TFAM. Noteworthy that HIF-1 expression was significantly higher in diapause-destined pupal brains than in non-diapause-destined pupal brains, suggesting a critical role of HIF-1-c-Myc-TFAM signaling in regulating insect diapause and/or lifespan extension (Lin et al., 2016).

## HIF-1-Mediated Signaling Facilitates Neuroprotection Upon Ischemic Injury

Regulation of the expression and activity of HIF-1 signaling represents a promising tool for ameliorating hypoxic brain damage. Several approaches to modulating HIF-1 complex activity have already been established. One widely used method for HIF-1 induction is the pharmacological blockade of enzymes involved in HIF-1α subunit degradation under normoxic conditions. PHD blockers are most commonly used for this purpose (Siddiq et al., 2005; Li et al., 2016; Barteczek et al., 2017). Deletion of the HIF suppressor prolyl-4-hydroxylase 2 (PHD2) leads to increased HIF-1α levels, which boosts cellular expression of EPO and VEGF. Suppression of PHD2 also increases neurogenesis in the hippocampus, and improves cognitive functions in mice suffering from chronic hypoperfusion of the brain. However, no changes were observed in the local density of blood capillaries, morphology of dendritic spines, or expression of genes associated with synaptic plasticity in the hippocampus (Gruneberg et al., 2016; Li et al., 2016). The use of PHD blockers, most of which are iron chelators, also results in the inhibition of cell death by ferroptosis, which may mediate their neuroprotective properties (Speer et al., 2013).

Another approach is the physiological induction of HIF-1α accumulation using short hypoxic preconditioning prior to acute hypoxia (Gu et al., 2008; Wu et al., 2020). Hypoxic preconditioning reportedly increases systemic resistance to ischemic brain damage, and reduces neuronal apoptosis by enhancing HIF-1α expression (Snigdha et al., 2012). Similar effects were obtained in zebrafish (Danio rerio), where pre-exposure of larvae to hypoxia increased hypoxia tolerance in adult fish (Mandic et al., 2020). Mandic et al. (2020) also demonstrated that in zebrafish, such improved hypoxia tolerance after pre-conditioning as well as general tolerance to hypoxia are mediated by HIF-1.

Activation of the HIF-1α signaling pathway also seems to be related to the modulation of neuronal apoptosis, although studies describing effects of the HIF-α complex on apoptosis have shown contradictory results. Overexpression of HIF-1α can lead to increased expression of some genes, facilitating cellular resistance against apoptosis caused by hypoxia or lack of nutrients (Akakura et al., 2001; Yang S. et al., 2017). For example, HIF-1α subunits are involved in regulating the activities of many members of the Bcl-2 family (Fang et al., 2020). Antiapoptotic actions of HIF-1α include regulation of the anti-apoptotic factors Bcl-2 (Sasabe et al., 2005) and Mcl-1 (Liu et al., 2006; Palladino et al., 2012); induction of Bcl-xL (Chen et al., 2009; Menrad et al., 2010); and downregulation of the pro-apoptotic factors Bid, Bax, and Bak (Sasabe et al., 2005). HIF-1α has also been associated with the regulation of mitochondrial functions via direct interactions with hexokinases—including hexokinase II, which catalyzes the first stage of glycolysis and can suppress apoptosis by binding to the voltage-dependent anion channel (VDAC) on the mitochondrial membrane (Pastorino and Hoek, 2008). Hexokinase II expression is controlled by HIF-1 (Semenza, 2001; Yasuda et al., 2004). Moreover, Bax- and Bak-independent induction of hexokinase II by HIF-1α can be critically involved in the initiation of a unique bioenergetic state of aerobic glycolysis, termed the Warburg effect, which is tightly associated with apoptosis inhibition (Lambert et al., 2010; Wolf et al., 2011).

On the other hand, HIF1α can also trigger p53-induced apoptosis via different mechanisms. Direct protein–protein interaction is proposed as the main mechanism leading to hypoxia-induced p53 stabilization. Two *in vitro* studies provide evidence suggesting a physical interaction between p53 and the oxygen-dependent degradation (ODD) domain of HIF-1α. Moreover, both *in vitro* and *in vivo* experiments have revealed interactions between HIF-1α and the modulator of p53 function Mdm2 (Roe et al., 2006).

Efforts to achieve neuroprotection have utilized molecules that negatively modulate the expression and/or stability of the HIF-1α subunit. Neuregulin-1 (NRG1) regulates neural cell differentiation (Mei and Xiong, 2008), neuronal growth and migration (López-Bendito et al., 2006; Mahar et al., 2016), neurotransmission (Neddens et al., 2009), and synaptic activity (Mei and Xiong, 2008; Mei and Nave, 2014). This protein can protect neurons in ischemic stroke and under oxidative stress (Xu et al., 2017; Kim et al., 2020; Wang et al., 2021). NRG1 weakens HIF-1α accumulation, inhibits HIF-1α nuclear localization, and increases and regulates neuronal hypoxic dysfunction. The neuroprotective effects of NRG1 appear to result from decreased HIF-1α-mediated stabilization of the p53 protein that initiates apoptosis (Yoo et al., 2019; Kim et al., 2020).

One important pathway contributing to the neuroprotective actions of HIF-1α involves increased expression of erythropoietin (EPO), which plays an essential role in the adaptation to hypoxia (Zhang et al., 2009; Li et al., 2020). EPO production is activated by hypoxia in the brain, uterus, and kidneys of mice (Chikuma et al., 2000). Moreover, exogenous EPO administration protects embryonic and postnatal hippocampal neurons from hypoxia-induced death, stimulates oligodendrogenesis, and reduces white matter damage (Iwai et al., 2010). EPO attenuates the inflammatory response by decreasing expressions of cyclooxygenase 2 and inducible NO synthase, and by suppressing microglial activation and inhibiting autophagy activation (Wu et al., 2018). Furthermore, increased HIF-1α expression in neurons and astrocytes leads to increased EPO expression, which inhibits neuronal apoptosis and thus facilitates the recovery of neurological functions (Rey et al., 2019; Li et al., 2020).

The neuroprotective effects of HIF-1α have been demonstrated in ischemic stroke, as well as in other settings. Traumatic brain injury (TBI) can lead to ischemic-hypoxic brain damage, and several *in vivo* studies show that TBI-induced cerebral hypoxia-ischemia plays a decisive role in the occurrence of various severe secondary brain injuries (Xu et al., 2012; Shu et al., 2016). After TBI, decreased oxygen delivery to nerve cells results in impaired glucose metabolism (Yamaki et al., 2018), which constitutes the main cause of neuronal apoptosis and neurological disorders after TBI (DeVience et al., 2017). Increased HIF-1α expression during TBI-induced hypoxia leads to increased expression levels of the glucose transporters GLUT1 and GLUT3, which enable greater glucose intake for hypoxic neurons (Wu et al., 2020). This improved neuronal energy supply can result in reduced neuronal death (Zhou et al., 2017; Wu et al., 2020).

Another important aspect of hypoxic damage is its possible involvement in neurogenesis. HIF-1α is an important regulator of hippocampal neurogenesis in postnatal organisms (Braun and Jessberger, 2014; Carrica et al., 2019). In mice, genetic inactivation of HIF-1α in nestin-positive hippocampal neural precursor cells leads to a pronounced decrease of neurogenesis, which results in deterioration of the spatial and contextual memory and the ability to learn. Notably, HIF-1α is not degraded in stem cells, including in neural stem cells of the subventricular zone (SVZ) of the hippocampus, even under normoxic conditions (Palomäki et al., 2013; Kim et al., 2019). Mechanistically, the self-renewal of stem cells can be regulated by the interplay between HIF-1α and the epigenetic regulator CBX7, and hypoxia leads to increased CBX7 expression through HIF-1α activation. Upon upregulation, CBX7 plays an essential role in enhancing the proliferation of neuronal progenitor cells during the postischemic period (Chiu et al., 2019). Overall, HIF-1α signaling is an important modulator for maintaining neurogenesis in the adult brain, and is thus a potential therapeutic target for the treatment of multiple neurodegenerative brain disorders.

## Adverse Effects of HIF-1α Signaling in Ischemia

As mentioned above, HIF-mediated signaling modulates the expressions of multiple target genes, some of which can evoke pathological processes (Sheldon et al., 2009; Barteczek et al., 2017). HIF-1 activation during the early acute phase of the hypoxic response triggers a cascade of adverse cerebral events that are associated with pentose phosphate pathway suppression (Vetrovoy et al., 2019). Under oxygen-glucose deprivation, glucose oxidation via the pentose phosphate pathway terminates oxidative stress development through modulation of redox homeostasis and the antioxidant system of the cell (Fernandez-Fernandez et al., 2012; Sun et al., 2017; Vetrovoy et al., 2019). During hypoxia, HIF-1 also strongly upregulates expression of the gene encoding the Na^+^-dependent chloride transporter NKCC1 (Yang et al., 2019). In recent studies in rat models of stroke and TBI, NKCC1 expressed in brain endothelial cells appears to be a critical mediator of edema formation and/or progressive secondary hemorrhage (Simard et al., 2010).

Experimental evidence shows that blockade of HIF-1α expression during early stages of hypoxia can contribute to increased neuronal viability both *in vitro* and *in vivo* (Cheng et al., 2014; Barteczek et al., 2017). Supporting this view, a recent study demonstrated the positive effect of using clinically relevant doses of caffeine to suppress the hypoxia-induced accumulation of HIF-1α (Li et al., 2019). Mice with double knockout of Hif-1α/Hif-2α exhibit significantly reduced expressions of the pro-apoptotic genes Bnip3, Bnip3L, and Pmaip1 (Barteczek et al., 2017). This resulted in a decreased rate of cell death, and reduced cerebral edema at 24 h after occlusion of the middle cerebral artery. Interestingly, this effect disappeared after 72 h of reperfusion. Accordingly, compared to wild-type animals, those with Hif-1α/Hif-2α deficiency exhibited preserved neurological status and sensorimotor functions on the first day after ischemia/reperfusion, but more severe symptoms after 72 h. This deterioration was accompanied by increased neuronal apoptosis and decreased angiogenesis. A similar short-term positive effect was observed at 24 h after ischemia/reperfusion after pharmacological inhibition of HIF-1 using 2ME2 (Cheng et al., 2014). Detailed molecular analysis revealed that 2ME2 treatment resulted in decreased levels of cleaved caspase-3; transcription factor NF-κB phospho-p65; phosphorylated kinase JNK1, 2/3; and total kinase JNK1, 2/3 (Cheng et al., 2014).

Other studies indicate that HIF-1α inhibition could be useful in later stages of hypoxia. Chen et al. (2017) showed that under chronic hypoxic conditions, HIF-1α levels significantly increased for more than 7 days, and HIF-1α knock-out significantly decreased neuronal apoptosis. However, other investigations have shown that agents that stimulates HIF-1α stabilization and nuclear translocation in early stages of hypoxia exert neuroprotective effects (Harms et al., 2010; Ryou et al., 2015).

HIF-1 can also be directly involved in the initiation of apoptosis. HIF-1α controls the gene encoding the Nip3 protein, a pro-apoptotic member of the Bcl-2 family (Bruick, 2000; Sowter et al., 2001). As mentioned above, under hypoxia, the pro-apoptotic protein p53 becomes stabilized in a manner that is dependent on hypoxia-induced HIF-1α accumulation (An et al., 1998; Krick et al., 2005). Due to the direct interaction between p53 and HIF-1α, decreased levels of transcriptionally active HIF-1 can lead to reduced HIF-1-dependent transcription of genes that prevent hypoxic damages (Wang et al., 2018; Madan et al., 2019; Wang et al., 2019). Notably, the impact of HIF-1 on apoptosis induction also depends on the severity of hypoxia. In mild hypoxia, HIF-1 has a rather protective effect due to the induced expressions of various anti-apoptotic proteins. In contrast, severe hypoxia (anoxia) leads to cell death, which is at least partly caused by HIF-1α-mediated p53 stabilization (Levine, 1997; Suzuki et al., 2001).

## Role of HIF-1α in Neurodegenerative Diseases

### Alzheimer’s Disease

Alzheimer’s disease (AD) is a severe neurodegenerative disease characterized by a complex etiology and different manifestation times. It is among the most common neurodegenerative diseases, affecting more than 15% of people aged 65 and over, and about 50% people over 85 years of age (Bonda et al., 2011). AD is characterized by two main histological and biochemical features: accumulation of amyloid beta-peptide (Aβ) in the brain, and the presence of neurofibrillary tangles comprising hyperphosphorylated tau protein. Amyloid beta-peptide exists in various lengths and in several forms, including globular, fibrillar, and oligomeric (Vinters, 2015).

The paradigm causally connecting cerebral hypoxia and AD is increasingly supported by experimental data. For example, cardiovascular risk factors are strongly correlated with sporadic AD (Leszek et al., 2020). Another significant risk factor for sporadic AD is TBI, especially chronic traumatic encephalopathy (Van Den Heuvel et al., 2007; Stein and Crary, 2020). AD is characterized by decreased blood flow in the brain and dysfunction of the neurovascular unit, which likely causes impaired oxygenation (Ahmad et al., 2020; Yu et al., 2020). Recent research also suggests that physical exercise can reduce AD risk by improving the brain’s oxygen supply (De la Rosa et al., 2020). Patients with Alzheimer’s disease have been shown to have reduced levels of HIF-1α as well as glucose transporters and a decreased rate of aerobic glycolysis in the brain. Decreased HIF-1a levels are associated with increased tau protein phosphorylation and neurofilament formation (Merelli et al., 2018). In addition, neurodegeneration progression is coupled with an increased production of reactive oxygen species leading to the development of oxidative stress and inflammation. These process can results in reduced expression of genes necessary to maintain the nerve cell viability and synaptic transmission, including HIF1 gene. Moreover, moderate production of mitochondrial ROS regulates PHD activity and causes stabilization of the HIF-1 complex.

**FIGURE 2:**
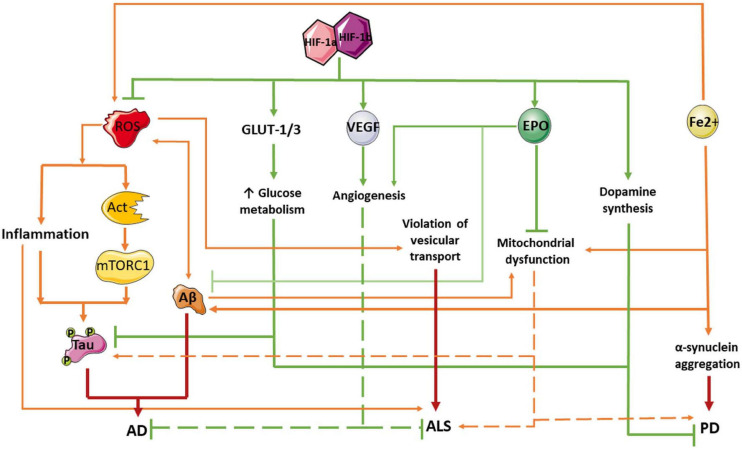
Protective role of HIF-1 pathway in neurodegeneration pathogenesis. Red arrows indicate negative effects and green lines and arrows mean positive effects. Aβ, amyloid beta; AD, Alzheimer’s disease; AKT, the serine-threonine protein kinase AKT; ALS, amyotrophic lateral sclerosis; EPO, erythropoietin; GLUT-1/3, facultative glucose transporter 1 or 3; HIF, hypoxia-inducible factor; mTORC1, mammalian target of rapamycin complex 1 or mechanistic target of rapamycin complex 1; PD, Parkinson’s disease; ROX, reactive oxygen species; Tau, Tau protein; VEGF, vascular endothelial growth factor.

Since multiple studies show a neuroprotective effect of the HIF-1α signaling pathway, stabilization of HIF-1 might be a promising therapeutic target for the treatment of neurodegenerative disorders. Pharmacological activation of HIF-1 can have a neuroprotective effect in AD, and thus might be used in therapy (Guo et al., 2015, 2017; Ashok et al., 2017; Merelli et al., 2018). Cognitive decline and AD progression can be slowed by increasing HIF-1 activity and/or increasing the expression of HIF-1 target genes involved in glycolysis or capillary blood supply regulation (Iyalomhe et al., 2016; Figure 2). For instance, administration of lactoferrin has been shown to promote the non-amyloidogenic processing of the amyloid precursor protein, resulting in an improvement of spatial learning and memory in APP/PS1 mice (Guo et al., 2017). Lactoferrin induces ERK-mediated activation of the ADAM10 metalloprotease via the HIF-1α pathway. Cultured hippocampal neurons transduced with an adenoviral vector encoding HIF-1α exhibited significantly reduced levels of apoptosis induced by amyloid beta-peptide (Chai et al., 2014). Moreover, several clinical trials have demonstrated that the administration of HIF-1 inducers can evoke neuroprotective effects in AD. Among others, such inducers include the multi-target iron chelating compound M30. Prolonged use of M30 increased the level of HIF-1α and the expression of its target genes involved in glycolysis, including aldolase A, enolase-1, and glucose transporter-1 (Glut-1) in the frontal cortex of APP/PS1 mice and improved their cognitive function (Mechlovich et al., 2014). In addition, in clinical trials in AD patients, the HIF-1 inducer deferoxamine (DFO) has reportedly slowed cognitive decline (Zhang et al., 2011). However, it must be noted that HIF-1 signaling can facilitate amyloidogenic processing of the amyloid-beta precursor protein, leading to an increased risk of AD in people with ischemia (Zhang X. et al., 2007; Ogunshola and Antoniou, 2009; Lee H.J. et al., 2016; Kim et al., 2017). For example, HIF-1 can promote BACE1-induced enhancement of Ab, thus contributing to AD progression (Lee H.J. et al., 2016; Guo et al., 2017). Moreover, in SK-N-MC cells, HIF-1α activates Aβ production via the Akt-mTOR-HIF-1α and Akt-NF-κB pathways (Kim et al., 2017). Thus, the role of HIF signaling in the development of AD-related neurodegeneration is rather controversial, and further research is required.

### Parkinson’s Disease

Parkinson’s disease (PD) is another widespread age-related neurodegenerative disorder associated with motor impairment (Rodriguez et al., 2015). Parkinson’s disease affects 0.3% of the world’s population, and 1–3% of people over 65 years of age (Tysnes and Storstein, 2017). The main characteristic of this disease is the progressive loss of dopaminergic neurons. During the initial stages of PD, this occurs in the substantia nigra region of the basal ganglia, bulb, and dorsal nucleus of the vagus nerve. Throughout PD progression, other brain regions become involved, including the pons, medulla, midbrain, mesocortical regions, and neocortex. Another typical feature of PD is the accumulation of α-synuclein’s Lewy bodies (LB) in the neuronal soma (Kalia and Lang, 2015; Shahmoradian et al., 2019). Although the molecular mechanisms underlying PD are not completely understood, there is strong evidence that its pathogenesis is significantly influenced by mitochondrial dysfunction and oxidative stress (Hauser and Hastings, 2013; Burbulla et al., 2017). PD is associated with decreased numbers of mitochondria and damage to the mitochondria structure, as well as with decreased activity of mitochondrial respiratory chain complex I, which may be linked to the actions of α-synuclein (Santos et al., 2014; Di Maio et al., 2016). At present, PD therapy mainly involves pharmacological dopamine substitution or increasing dopamine production. However, these treatments provide only symptomatic effects, without neuroprotection. Therefore, current research efforts are underway to investigate promising new therapeutic strategies (Jankovic and Tan, 2020; Mao et al., 2020).

It has been shown that HIF-1α deficiency may play an important role in the pathogenesis of PD. Indeed, HIF-1 is essential for differentiation and survival of dopaminergic neurons. A decrease in the HIF-1 expression leads to neuronal death during the development of Parkinsonism. Therefore, an increase in the expression of HIF-1α might be a promising therapeutic approach for PD patients (Mehrabani et al., 2020). HIF-1 complex activation has neuroprotective effects in both *in vitro* and *in vivo* PD models, and the underlying mechanisms appear to be related to HIF-1-mediated expression of the erythropoetin (EPO) and vascular endothelial growth (VEGF) genes (Figure 2; Schofield and Ratcliffe, 2004; Semenza, 2014; Strowitzki et al., 2019). Feng et al. (2014) demonstrated that the neuroprotective effect of neuropeptide orexin-A in an *in vitro* PD model could be explained by HIF-1α induction, followed by the expression of its downstream targets, including VEGF and EPO. Systemic EPO administration also modulates long-term synaptic plasticity (Almaguer-Melian et al., 2016), has antioxidant effects when injected into the striatum (but not into the substantia nigra), and reduces inflammatory responses (Thompson et al., 2020). Therefore, HIF-mediated regulation of EPO can be considered a promising approach for PD therapy.

Modulation of HIF-1 signaling can also protect neurons from oxidative stress, which is a damaging factor involved in PD (Figure 2). One mechanism for this protective effect is the inhibition of proteins that mediate HIF-1α degradation via the ubiquitin-dependent proteasome pathway—for example, through orexin-mediated inhibition of the von Hippel-Lindau protein (vHL) or E3 ubiquitin ligase expression (Feng et al., 2014; Liu et al., 2018, 2020). Moreover, HIF-specific prolyl hydroxylases can be inhibited using low-molecular-weight inhibitors or interfering RNA (Johansen et al., 2010; Li et al., 2018; Aimé et al., 2020; Mehrabani et al., 2020). Such studies have been conducted in multiple cellular PD models, including PC12 cells (Johansen et al., 2010), SH-SY5Y cells (Li et al., 2018; Aimé et al., 2020; Mehrabani et al., 2020), and primary cultures of nerve cells from mice treated with the neurotoxin 6-OHDA (Johansen et al., 2010; Zhang et al., 2020). Application of the low-molecular-weight HIF inhibitor prolyl hydroxylase leads to increased expression and activation of tyrosine hydroxylase, thereby enhancing dopamine synthesis and release. In animal models, low-molecular-weight prolyl hydroxylase domain (PHD) inhibitors reduce the loss of tyrosine hydroxylase-positive neurons of the substantia nigra, and attenuate behavioral disturbances in mice. HIF PHD inhibition also leads to amelioration of mitochondrial functions. In cellular PD models, PHD inhibitors normalize the mitochondrial membrane potential and the rate of mitochondrial oxygen consumption, and reduce the production of reactive oxygen species (Zhang et al., 2018). Among PHD inhibitors, compounds that do not bind iron show greater promise for the treatment of PD and others neurodegenerative disorders, since chronic administration of chelators can lead to restless legs syndrome (RLS) (Earley et al., 2014) or anemia (White et al., 2005).

### Amyotrophic Lateral Sclerosis

Amyotrophic lateral sclerosis (ALS), also known as Lou Gehrig’s disease or motor neuron disease, is a severe neurodegenerative disorder characterized by the death of the upper and/or lower motor neurons of the motor cortex and spinal cord (Van Es et al., 2017). ALS affects about 0.03% of the world’s population, and death usually occurs 3–4 years after the initial diagnosis (Longinetti and Fang, 2019). ALS causes muscle weakness and atrophy, which affects all of the muscles, ultimately resulting in death due to respiratory failure (Lechtzin et al., 2018). ALS pathogenesis is characterized by pronounced vascular changes and blood flow disturbances, accompanied by decreased expressions of EPO and VEGF (Miyazaki et al., 2011; Pronto-Laborinho et al., 2014), which result in tissue hypoxia. Decreased oxygen levels in tissues lead to excessive ROS production and cell death (Tafani et al., 2016). Therefore, an impaired adaptive response to hypoxia is considered a likely reason for motor neuron death in ALS (Kim et al., 2013; Yamashita et al., 2020). Accordingly, in animal models of ALS, increasing blood flow in the hypoxia-affected areas of the spinal cord has protective effects against neurodegeneration (Zheng et al., 2004; Tada et al., 2019).

Interestingly, HIF-1α expression is elevated before the onset of clinical ALS symptoms, and is then significantly decreased in later disease stages (Nomura et al., 2019). Dysregulation of HIF-1α expression, with consequent disruption of the downstream pathway involved in anti-hypoxic response, can augment the motor neuron degeneration in ALS (Figure 2; Moreau et al., 2011; Nagara et al., 2013). Several *in vitro* and *in vivo* studies suggest the neuroprotective effects of HIF activation in ALS. In a mouse model of ALS, facilitation of HIF-1α signaling reduced hypoxic damage, resulting in neuroprotective and anti-inflammatory effects, and reduced motor neuron degradation (Nomura et al., 2019). Another study demonstrated that the inhibitor of PHD, Fumaric acid esters (FAE), causes the activation of HIF-1α in astrocytes and the subsequent production of VEGF and GLUT. It led to an increase in neuronal survival in the *in vitro* ALS model (Wiesner et al., 2013). Contrary to these results, Tada et al. (2019) recently demonstrated that reducing HIF-1α expression using the prostacyclin analog ONO-1301-MS increased the survival rate and motor functions in an animal model of ALS. These contradictory results indicate an urgent need for further in-depth studies of the role of HIF in ALS pathogenesis.

### Huntington’s Disease

Huntington’s disease (HD) is an autosomal dominant neurodegenerative disorder that leads to deterioration of physical and mental abilities. HD is characterized by chorea, dystonia, impaired coordination of movements, and decreased cognitive functions (McColgan and Tabrizi, 2017). This disease affects up to 0.15% of the world’s population in different countries (McColgan and Tabrizi, 2017; Cheng et al., 2020). HD is caused by the expansion of a polymorphic CAG repeat in exon 1 of the huntingtin (HTT) protein gene located on the short arm of chromosome 4p16.3. This mutation results in an abnormally long expansion of polyglutamine in the HTT protein, which interferes with protein folding, thus facilitating its aggregation and accumulation and resulting in neurodegeneration (Carroll et al., 2015). The CAG repeat number is inversely related to the age at the initial HD manifestation (Squitieri et al., 2001).

The destructive effect of HTT mutation can be partly explained by mitochondrial dysfunction and fragmentation, and energy metabolism impairment (Yano et al., 2014; Zheng et al., 2018). Therefore, various methods of maintaining mitochondrial function are currently considered potential strategies for reduction of neurodegeneration in HD patients (Yang et al., 2020; Burtscher et al., 2020). Among such methods, the modulation of molecular cascades involved in cellular adaptation to hypoxia represents a promising therapeutic strategy for slowing HD development, which is supported by increasing experimental evidence. For instance, in a mouse model of HD, treatment with low-molecular-weight HIF PHD inhibitors resulted in the diminution of symptoms and slowing of disease progression. HIF PHD inhibition also protected cortical neurons from cytotoxicity induced by a complex II inhibitor 3-nitropropionic acid. Notably, cortical neuron viability was correlated with increased expression of the VEGF gene, but not with increased expression of the PGC-1α gene (Niatsetskaya et al., 2010).

### Multiple Sclerosis

Multiple sclerosis (MS) is a chronic, autoimmune, neurodegenerative disorder of the central nervous system. The clinical symptoms most commonly manifest between 20 and 40 years of age, leading to a fast progression of neurological, physical, and cognitive disability in young adults (Gilmour et al., 2018). The clinical symptoms of MS include sensory, motor, and cognitive impairment; visual and speech impairment; increased fatigue; and pain (Compston and Coles, 2008; Garg and Smith, 2015). MS is a disease of unclear etiology, which arises from an interplay between multiple non-genetic and genetic risk factors. The clinical onset is influenced by a combination of genetic predisposition and multiple trigger factors, such as UV radiation, vitamin D, viral infections, smoking, obesity, and other environmental factors (Handel et al., 2010; Belbasis et al., 2019). At the cellular level, MS is characterized by pronounced demyelination, axonal degeneration, neuronal inflammation, and glial activation. MS treatment includes three main strategies: treatment of relapse with anti-inflammatory drugs, symptomatic treatment, and the use of drugs that change the disease course (disease-modifying therapies; DMTs) (DeLuca et al., 2020; Goldschmidt and McGinley, 2021).

One important factor that predisposes a patient to MS and promotes MS is brain hypoxia resulting from decreased cerebral blood flow, impaired microcirculation, and local formation of toxic metabolites that inhibit mitochondrial activity (Aboul-Enein and Lassmann, 2005; Yang and Dunn, 2018). Hypoxia is an important pathogenic component for all forms of MS in the early stages, and it is assumed that hypoxia can act as a trigger in MS pathogenesis (Yang and Dunn, 2018; Halder and Milner, 2020). Hypoxia can boost inflammatory processes that, in turn, facilitate MS development. Moreover, inflammation can provoke the development of hypoxia, generating a vicious circle (Yang and Dunn, 2018; Halder and Milner, 2020). Indeed, inflammatory processes are tightly associated with increased oxygen consumption, which leads to local hypoxia, and subsequently to local activation of HIF-1. One possible strategy for interrupting this pathological interplay thus involves the targeted modulation of molecular cascades activated upon hypoxia, including the HIF signaling pathway. However, the presently available data regarding the effects of HIF-1 in MS are rather contradictory. Some studies show that HIF-1 stabilization can slow down MS progression (Yao et al., 2008; Sun et al., 2010; Deng et al., 2016; Guan et al., 2017). On the other hand, some evidence suggests that HIF-1 might be involved in T-cell activation, thus contributing to a more severe disease course (De Riccardis et al., 2015; Deng et al., 2016).

## Concluding Remark

In this review, we provided an overview of the main molecular cascades activated by the HIF-1α in response to cerebral hypoxia. Increased expression and stabilization of HIF-1α enhance angiogenesis and erythropoiesis, activate anti-apoptotic cascades, and thus can represent a promising therapeutic strategy in the treatment of cerebral ischemia and multiple neurodegenerative disorders, including AD, PD, ALS, and MS. However, it should be taken in consideration that although multiple studies demonstrated neuroprotective effects of HIF-1α stabilization, a couple of evidence indicate that HIF-1α inhibition might have a positive effect in several neurodegenerative disorders. Moreover, an impact of the HIF signaling strongly depends on the severity of hypoxic damage and can also be harmful. Thus, there is a clear need for further studies to better define the criteria for development and using highly selective modulators of the HIF signaling pathway.

According to the reviewed literature, the use of inhibitors of PHD activity to stabilize HIF-1α levels appears to be a promising therapeutic approach. One of the main directions of further research should thus be the choice of the optimal time for the administration of these inhibitors during the development of ischemic damage. Such strategy will optimize the effectiveness of the inhibitors and help to avoid side effects. On the other hand, when searching for HIF-1-based approaches to the therapy of neurodegenerative diseases, one should consider that chronic inhibition of PHD can lead to the local hyperoxia in previously hypoxic areas followed by excessive angiogenesis and a change in the status of progenitor cells. Of note, there are currently very few studies on the effect on the HIF-mediated signaling in modulating the resistance of the nervous tissue to hypoxia, correct mitochondrial dysfunction and maintain glucose metabolism in such Huntington’s disease, MS, and ALS. Undoubtedly, this area requires more profound research. Finally, despite a number of important controversial points requiring additional research, it is obvious that new therapies associated with the activation of the HIF cascade look very promising.

## Author Contributions

EM, MS, and EP wrote the manuscript with input from all authors. EM, EP, and MV supervised the project, conceptualized the original idea, and were in charge of the overall direction. All authors read and approved the final manuscript.

## Conflict of Interest

The authors declare that the research was conducted in the absence of any commercial or financial relationships that could be construed as a potential conflict of interest.

## Publisher’s Note

All claims expressed in this article are solely those of the authors and do not necessarily represent those of their affiliated organizations, or those of the publisher, the editors and the reviewers. Any product that may be evaluated in this article, or claim that may be made by its manufacturer, is not guaranteed or endorsed by the publisher.
